# A Subjective Assessment of the Prevalence and Factors Associated with Poor Sleep Quality Amongst Elite Japanese Athletes

**DOI:** 10.1186/s40798-018-0122-7

**Published:** 2018-02-26

**Authors:** Masako Hoshikawa, Sunao Uchida, Yuichi Hirano

**Affiliations:** 1 Department of Sport Research, Japan Institute of Sport Sciences, 3-15-1 Nishigaoka, Kita-ku, Tokyo, 115-0056 Japan; 2 Faculty of Sport Sciences, Waseda University, Mitakajima, Tokorozawa, Saitama, Japan; 3 Faculty of Sports and Health Studies, Hosei University, 4342 Aihara-Cho, Machida, Tokyo, 194-0298 Japan

**Keywords:** Elite athletes, Sleep quality, Questionnaire study

## Abstract

**Background:**

The amount, quality, and timing of sleep are considered important for athletes’ ability to train, maximize training responses, and recover. However, some research has shown that elite athletes do not obtain sufficient sleep. Based on this background, researchers recently started to assess and manage sleep in elite athletes.

The purpose of this study was to clarify the prevalence of poor sleep quality and its associated factors amongst elite Japanese athletes.

**Methods:**

Eight hundred and ninety-one candidates for the 17th Asian Games Incheon 2014, who were over 20 years old, participated in this study. They completed a questionnaire that included the Pittsburgh Sleep Quality Index (PSQI), Epworth Sleepiness Scale, two-question case-finding instruments, and a checklist for sleep hygiene. Data from 817 of the 891 athletes (91.7%) with no missing values were analyzed.

**Results:**

The mean time in bed was 7 h and 29 min. Two hundred and twenty-nine (28.0%) athletes showed a PSQI global score above the clinical criteria. A multiple logistic analysis revealed that sleep quality was significantly associated with five factors: “time in bed,” “eating breakfast every morning,” “avoiding the use of electronic devices (PC, smartphone, etc.) just before bedtime,” “depressive mood”, and “not thinking about troubles while in bed.” Forty percent of athletes reported they had been informed by someone about “snoring loudly” and/or “leg twitching or jerking during sleep.”

**Conclusions:**

The results of this study demonstrate that 28% of the athletes showed the PSQI score above the cutoff for poor sleep quality (> 5.5), which suggests that there may be a high prevalence of poor sleep quality in this population of athletes. To improve athletes’ sleep, the five factors associated with sleep quality should be emphasized in athletes’ sleep education. Furthermore, in medical evaluations of athletes, it may be desirable to include screening for sleep disorders.

## Key Points

- Twenty-eight percent of elite Japanese athletes showed a PSQI global score above the cutoff for poor sleep quality.
- The PSQI global score above the cutoff for poor sleep quality was associated with five factors: “time in bed,” “eating breakfast every morning,” “avoiding the use of electronic devices (PC, smartphone, etc.) just before bedtime,” “depressive mood,” and “not thinking about troubles in bed.”
- Forty percent of the athletes had been informed by someone about “snoring loudly” and/or “leg twitching or jerking during sleep.”

## Background

As represented by the Olympic motto “faster, higher, stronger,” Olympic athletes must keep improving their athletic performance. To achieve this, they increase their frequency, volume, and intensity of training. Such an increase in the training load is often successful, and their performance improves. Since the training load will be compromised during periods of fatigue, fatigue management is very important for a successful increase in the load [1]. If athletes cannot maintain a favorable condition, it may be difficult to train as scheduled and to achieve good results.

Sleep is arguably one of the forms of recovery and regeneration available to elite athletes. The amount, quality, and timing of sleep are important for athletes’ ability to train, maximize training responses, and recover. However, some research has shown that elite athletes do not obtain an adequate amount of sleep and that the prevalence of poor sleep quality is high (> 20%) [2–4]. Based on this background, researchers recently started to assess and manage sleep in elite athletes [3, 4].

The amount, quality, and timing of sleep in elite athletes may be affected by many factors, such as training-practice schedules [3, 4], training or exercise volumes [5–9], match schedules [3, 10, 11], nervousness due to competitions [12–15], and sleep disorders [16–20]. Inappropriate sleep hygiene [21–25] is also known to influence the amount and quality of sleep; however, the present status of sleep hygiene in elite athletes has not been studied extensively.

Strictly speaking, factors that affect sleep quality may depend on the individual athletes. However, clarifying general factors correlated with sleep quality may be helpful for the sleep education of athletes.

The Pittsburgh Sleep Quality Index (PSQI) is a widely used standardized measure to assess subjective sleep quality [26]. The PSQI has been applied to both athletes and general populations. Fietze et al. reported that 12 of 24 German ballet dancers showed PSQI global scores above the cutoff level (> 5.5) [27]. In the Netherlands, Knufinke et al. reported that the mean PSQI global score in athletes was 4.61 and that 40 of 98 athletes showed a PSQI global score above the cutoff level [28]. In Japan, we studied 310 elite Japanese athletes and reported that the mean PSQI global score was 4.8, and 95 of the 310 athletes showed global scores above the cutoff level [29].

In this study, we defined sleep quality as being poor if the PSQI global score was above the cutoff level. A logistic regression analysis was performed to examine relevant factors, such as body mass index (BMI), some clinical information, mood, training-practice schedules, frequencies of overseas trips, sleep schedule, and sleep hygiene, and their association with poor sleep quality. The purpose of this study was to clarify the prevalence of poor sleep quality and its associated factors amongst elite Japanese athletes.

## Methods

### Participants

A total of 891 candidates for the 17th Asian games Incheon 2014, who were over 20 years old, participated in this study. The survey started on February 20 and ended on July 15, 2014. (The 17th Asian games Incheon 2014 was held between September 19 and October 4). Written informed consent was obtained after explaining the purpose, procedure, and possible risks of this study. This study was approved by the Japan Institute of Sports Sciences Ethics Committee.

Data from 817 (449 males and 368 females) of the 891 athletes (91.7%) with no missing data were analyzed.

### Questionnaire

The athletes were asked to complete the questionnaire after measuring their heights and weights. The questionnaire consisted of questions to obtain general information, the PSQI [30], the Epworth Sleepiness Scale (ESS) [31], two-question case-finding instruments [32], and a checklist for sleep hygiene. The general information included sex, age, sport, training-practice schedules, napping habits, and frequency of overseas trips in the past 6 months. Training-practice schedules were ascertained on each day of the week. Training-practice times per week were calculated from the training-practice times on each day.

The PSQI consists of 19 self-rated questions. The athletes were asked about their bed time, sleep latency, and getting-up time, and the time in bed and sleep efficiency were calculated as part of the PSQI. Subjective sleep quality was evaluated based on the global score of the PSQI. In the Japanese version, a PSQI global score higher than 5.5 is considered to indicate poor sleep quality [33]. The PSQI contains five other questions that are not reflected in the global score and are rated by a bed partner or roommate [26, 30]. Since the athletes were not accompanied by a bed partner or roommate at the time of completing the questionnaire, the latter five questions were modified to be answered by the athletes themselves in this study as follows:

- Has any of your family, people living with you, or people who have stayed in the same room as you during a tour or other occasions made the following remarks to you?
  (A) You snore loudly
  (B) You take long pauses between breaths while asleep
  (C) Your legs twitch or jerk while asleep
  (D) You show episodes of disorientation or confusion during sleep
  (E) You show other types of restlessness while asleep

For each of the five items, athletes selected an appropriate answer from the following: “Not at all,” “Yes, sometimes,” and “Yes, frequently.” For the logistic regression analyses, “Yes, sometimes” and “Yes, frequently” were defined as “the athlete has the symptom.” For the logistic regression analyses, “the athlete has the symptom” was assigned the dummy variable “1,” and the absence of the symptom was assigned “0.”

Napping habits were clarified by asking three questions. Firstly, the athletes stated their frequency of napping by selecting one of five choices: no napping habits, less than once per week, 1–2 times per week, 3–5 times per week, and almost every day. Athletes with napping habits were requested to answer the second and third questions. In the second question, athletes stated their timing of starting to nap by selecting one of nine choices: before noon, eight time bands of every 1 hour from 12:00 to 19:00, and depends on case. Thirdly, athletes stated their duration of napping by selecting one of five choices: 30 min or less, 31 to 60 min, 61 to 90 min, more than 90 min, and depends on case. For the logistic regression analyses, “no napping habit” and “within 30 min” were assigned the dummy variable “0,” and the others were assigned “1.”

Daytime sleepiness was evaluated by the ESS score (Japanese version). An ESS score higher than 10.5 is considered to indicate excessive daytime sleepiness [31].

Two-question case-finding instruments for depression were used to evaluate each athlete’s mood [32]. The instruments consisted of two questions: (1) “During the past month, have you often been bothered by feeling down, depressed, or hopeless?” and (2) “During the past month, have you often been bothered by little interest or pleasure in doing things?” Athletes were requested to answer “yes” or “no” to each question. For Japanese adults, a sensitivity of 99% and a specificity of 60.5% have been reported if the respondent answers “yes” to either of the two questions. If the respondent answers “yes” to both questions, a sensitivity of 87.9% and a specificity of 81.4% have been reported [34]. For the logistic regression analyses, the answer “yes” to both questions was considered “the athlete feels a depressive mood.” For the logistic regression analyses, “the athlete feels a depressive mood” was assigned the dummy variable “1,” and its absence was assigned “0.”

**Table:** Sleep hygiene was assessed using the modified checklist reported by Tamura and Tanaka [35].

・Being bathed in sunlight after getting up	
・Eating breakfast every morning	
・Not taking a nap after 15:00	
・Not going out to bright places after 21:00	
・Avoiding the use of electronic devices just before bed time	
・Not drinking alcohol to induce sleep	
・Not thinking about troubles in bed	
・Keeping the bedroom quiet and comfortable	
・Using a comfortable mattress and bedclothing	
・Preventing an irregular getting up time (within 2 h)	

Each question had three possible replies: “I already do so,” “I may be able to, but do not already do so,” and “I think it is difficult to do so.” For the logistic regression analyses, “I may be able to, but do not already do so” and “I think it is difficult to do so” were defined as “the athlete does not already do so.” For the logistic regression analyses, “the athlete does not already do so” was assigned the dummy variable “1,” and the other was assigned “0.”

The athletes were asked about the frequency of overseas trips in the past 6 months.

The questionnaire took approximately 10 min to complete.

### Statistical Analysis

For describing general information, data are expressed as the mean ± SD. Statistical comparisons between sexes were conducted by the independent *t* test for continuous variables and by chi-square tests and residual analyses for discrete variables.

In addition, the results of training-practice schedules, sleep schedule, the PSQI, the ESS, and clinical information were categorized within each variable, and then the number and percentages of athletes were described in the categories.

To explore factors associated with poor sleep quality (PSQI global score > 5.5), logistic regression analyses were performed using the following independent variables: age, sex, BMI, frequency of overseas expeditions, training-practice schedules, sleep schedules, clinical information, depressive mood, and sleep hygiene. Initially, we examined these variables using a univariate analysis. Then, a multiple logistic regression analysis was performed using the variables showing significant correlations in the univariate analysis (*p* < 0.05). For the continuous variables, the results were used for the logistic regression analyses. For the variables regarding clinical information, depressive mood, and sleep hygiene, dummy variables were used for the logistic regression analysis. Wald statistics were used to examine the significant odds ratios generated by the regression analyses. All analyses were performed using IBM SPSS 22 for windows. Significance was set at *p* < 0.05.

## Results

The athletes’ heights, body weights, and BMIs were as follows: 177.5 ± 8.7 cm, 76.7 ± 12.9 kg, and 24.2 ± 3.0 kg/m^2^ for males, and 165.0 ± 7.7 cm, 59.9 ± 8.7 kg, and 22.1 ± 2.6 kg/m^2^ for females, respectively (*p* < 0.001). The BMIs in 296 males (65.9%) were less than 25 kg/m^2^, and those in 153 males (34.1%) were equal to or greater than 25 kg/m^2^. The indexes in 333 females (90.5%) were less than 25 kg/m^2^, and those in 35 females (9.5%) were equal to or greater than 25 kg/m^2^.

The characteristics of the athletes’ daily schedules are presented in Table 1. If the athletes have several types of training-practice schedules, the earliest start and latest end times were adopted as representative. The statistical analysis revealed significant differences between the sexes in the latest training-practice end time (18:20 ± 2:00 for males and 18:36 ± 2:00 for females, *p* < 0.01) and the hours of training-practice per week (22 h 30 min ± 10 h 12 min for males and 24 h 36 min ± 12 h for females, *p* < 0.05), but not in the earliest training-practice start time (9:48 ± 2:42 for males and 9:30 ± 2:42 for females). The mean time in bed was 7 h and 29 min in total: 7 h and 37 min for males and 7 h and 20 min for females (*p* < 0.001). Two point four percent of males and 6.3% of females were considered short sleepers (time in bed < 6 h). For both males and females, more than half of the athletes have napping habits of once or more than once per week. Additionally, both males and females tend to prefer a nap duration of 31–60 min.

**Table 1:** The characteristics of daily schedules in elite Japanese athletes

		Male		Female	
		*N*	(%)	*N*	(%)
Earliest training-practice start time	Before or at 7:00	68	(15.5)	60	(16.6)
	7:01–8:00	16	(3.6)	26	(7.2)
	8:01–9:00	121	(27.5)	97	(26.9)
	9:01–10:00	152	(34.5)	120	(33.2)
	After 10:00	83	(18.9)	58	(16.1)
Latest training-practice end time	Before or at 18:00	261	(59.5)	152	(42.2)
	18:01–19:00	47	(10.7)	69	(19.2)
	19:01–20:00	47	(10.7)	69	(19.2)
	20:01–21:00	68	(15.5)	54	(15.0)
	After 21:00	16	(3.6)	16	(4.4)
Hours of training-practice per week	20 h or less than 20 h	190	(43.6)	144	(40.2)
	20.1–30 h	164	(37.6)	130	(36.3)
	30.1–40 h	66	(15.1)	53	(14.8)
	40.1–50 h	10	(2.3)	18	(5.0)
	More than 50 h	6	(1.4)	13	(3.6)
Number of training-practice session per week	1–5 times per week	88	(19.6)	70	(19.0)
	6–10 times per week	231	(51.4)	177	(48.1)
	More than 10 times per week	130	(29.0)	121	(32.9)
Usual getting up time	Before or at 6:00	100	(22.3)	103	(28.0)
	6:01–7:00	191	(42.5)	182	(49.5)
	7:01–8:00	118	(26.3)	68	(18.5)
	After 8:00	40	(8.9)	15	(4.1)
Usual time in bed	< 5 h	4	(0.9)	2	(0.5)
	≧ 5 and < 6 h	7	(1.6)	21	(5.7)
	≧ 6 and ≦ 7 h	142	(31.6)	132	(35.9)
	> 7 and ≦ 8 h	186	(41.4)	155	(42.1)
	> 8 h	110	(24.5)	58	(15.8)
Nocturnal sleep duration	< 5 h	3	(0.7)	7	(1.9)
	≧ 5 and < 6 h	19	(4.2)	36	(9.8)
	≧ 6 and ≦ 7 h	258	(57.5)	225	(61.1)
	> 7 and ≦ 8 h	144	(32.1)	90	(24.5)
	> 8 h	25	(5.6)	10	(2.7)
Frequency of naps	No habit	126	(28.1)	89	(24.2)
	Less than once per week	59	(13.1)	69	(18.8)
	1–2 times per week	99	(22.0)	67	(18.2)
	3–5 times per week	118	(26.3)	87	(23.6)
	Almost every day	47	(10.5)	56	(15.2)
Nap duration	≦ 30 min	63	(14.0)	53	(14.4)
	31–60 min	147	(32.7)	134	(36.4)
	61–90 min	75	(16.7)	50	(13.6)
	≧ 90 min	11	(2.4)	13	(3.5)
	Depends on case	27	(6.0)	29	(7.9)

Table 2 describes the mean time in bed in athletes specialized in each sport. Each mean value was calculated when the values were obtained from more than four athletes. The results show a time in bed of less than 7 h in males from five sports and in females from nine sports.

**Table 2:** The mean time in bed of athletes specialized in each sport

		Male		Female	
		h	(*n*)	h	(*n*)
Aquatics	Swimming	6.9	(12)	7.6	(12)
	Diving	7.0	(4)		
	Synchronized swimming			7.6	(11)
	Water polo	7.9	(15)	7.1	(15)
Archery		7.3	(4)	6.8	(5)
Athletics		7.5	(33)	7.4	(26)
Badminton		8.1	(11)	8.0	(11)
Baseball	Baseball	7.7	(30)		
	Softball			7.2	(16)
Basketball		8.0	(20)	7.6	(24)
Bowling		7.5	(5)	6.9	(5)
Boxing		7.9	(4)		
Canoe/kayak	Sprint	7.8	(9)	8.6	(4)
	Obstacle slalom	8.0	(4)		
Cricket				6.7	(13)
Cycling	Track	7.8	(11)	7.9	(8)
	Road race	8.5	(5)	7.6	(6)
	Mountain bike				
	Bicycle motocross				
Equestrian		7.4	(8)		
Fencing		7.6	(9)	7.4	(8)
Football		8.1	(21)	7.9	(19)
Golf		8.1	(4)		
Gymnastics	Artistic	8.3	(4)		
	Rhythmic				
	Trampoline	7.7	(8)		
Handball		7.8	(22)	7.6	(19)
Hockey		8.3	(25)	7.2	(18)
Judo		7.0	(12)	7.6	(8)
Kabaddi		6.3	(10)	6.1	(10)
Karate		8.1	(4)	6.1	(4)
Modern pentathlon		7.7	(9)	7.6	(6)
Rowing		7.3	(15)		
Rugby		7.9	(18)	7.0	(18)
Sailing		7.9	(6)	8.5	
Sepaktakraw		7.0	(13)	6.4	(9)
Shooting		7.2	(8)	6.7	(11)
Squash		6.3	(2)		
Table tennis		7.3	(4)	7.7	(7)
Taekwondo		6.6	(4)		
Tennis	Tennis	8.1	(5)	7.9	(5)
	Soft tennis	7.6	(5)	7.2	(4)
Triathlon		6.8	(5)	7.0	(7)
Volleyball	Volleyball	7.5	(24)	7.7	(20)
	Beach volleyball				
Weightlifting		7.3	(7)	6.7	(6)
Wrestling		7.4	(13)		
Wushu		7.4	(7)	6.8	(6)

The results of the PSQI and the ESS are presented in Table 3. Two-hundred and twenty-nine (28.0%) athletes, 111 (24.7%) males and 118 (32.1%) females, showed a PSQI global score above 5.5. The mean and SD of the PSQI global scores were 4.2 ± 2.1 for males and 4.7 ± 2.2 for females (*p* < 0.01). The PSQI sub-category scores indicated the presence of 34 (4.2%) athletes with a long sleep latency. The sub-category scores also indicated that 76 (9.3%) athletes had difficulty maintaining their sleep. Fifty (11.1%) males and 51 (13.9%) females showed a sleep efficiency lower than 85%. Two-hundred and sixty-five (32.4%) athletes, 112 (24.9%) males and 153 (41.6%) females, showed an ESS score above the cutoff level. The mean and SD of the ESS scores were 8.2 ± 4.0 for males and 9.7 ± 4.1 for females (*p* < 0.001). Overall, both the PSQI global and ESS scores were below the cutoff for 417 (51.0%) athletes. For 91 (11.1%) athletes, both scores were above the cutoff.

**Table 3:** Results of the Pittsburgh sleep quality index (PSQI) global and subcategories and Epworth Sleepiness Scale (ESS) scores

			Male		Female	
		Score	*N*	(%)	*N*	(%)
PSQI-global score		≤ 5.5	338	75.3	250	67.9
		> 5.5	111	24.7	118	32.1
PSQI-sub-category score	Self-rated sleep quality	0	50	11.1	40	10.9
		1	309	68.8	226	61.4
		2	87	19.4	100	27.2
		3	3	0.7	2	0.5
	Sleep latency	0	146	32.5	127	34.5
		1	185	41.2	145	39.4
		2	100	22.3	80	21.7
		3	18	4.0	16	4.3
	Sleep duration	0	169	37.6	100	27.2
		1	258	57.5	225	61.1
		2	19	4.2	36	9.8
		3	3	0.7	7	1.9
	Sleep efficiency	0	399	88.9	317	86.1
		1	43	9.6	42	11.4
		2	5	1.1	7	1.9
		3	2	0.4	2	0.5
	Sleep disturbance	0	163	36.3	108	29.3
		1	283	63.0	254	69.0
		2	3	0.7	6	1.6
		3	0	0.0	0	0.0
	Use of sleeping meds	0	434	96.7	362	98.4
		1	10	2.2	4	1.1
		2	1	0.2	1	0.3
		3	4	0.9	1	0.3
	Daytime dysfunction	0	254	56.6	170	46.2
		1	154	34.3	162	44.0
		2	33	7.3	33	9.0
		3	8	1.8	3	0.8
ESS-score		≤ 10.5	337	75.1	215	58.4
		> 10.5	112	24.9	153	41.6

Regarding clinical information, approximately 40% of the athletes had been informed by someone about “snoring loudly” and/or “leg twitching or jerking during sleep” (Table 4). Males were frequently described as loud snorers (*p* < 0.001) and taking long pauses between breaths (*p* < 0.01, Table 4). According to BMI, 4.7% of males and 1.2% of females in the categories with a BMI below 25 kg/m^2^ had been informed of long pauses between breaths, whereas those values were 12.3 and 11.4% in the categories with a BMI at or above 25 kg/m^2^, respectively. Leg twitching or jerking during sleep was remarked in 55.5% of females (*p* < 0.01).

**Table 4:** The frequency and distribution of responses to clinical questions

		Male		Female	
		*N*	(%)	*N*	(%)
Loud snoring	Not at all.	236	(52.6)	267	(72.6)
	Yes, sometimes.	167	(37.2)	86	(23.4)
	Yes, frequently.	46	(10.2)	15	(4.1)
Long pauses between breaths while asleep	Not at all.	419	(93.3)	361	(98.1)
	Yes, sometimes.	25	(5.6)	6	(1.6)
	Yes, frequently.	5	(1.1)	1	(0.3)
Legs twitching or jerking while asleep	Not at all.	251	(55.9)	164	(44.6)
	Yes, sometimes.	144	(32.1)	136	(37.0)
	Yes, frequently.	54	(12.0)	68	(18.5)
Episodes of disorientation or confusion during sleep	Not at all.	398	(88.6)	329	(89.4)
	Yes, sometimes.	48	(10.7)	36	(9.8)
	Yes, frequently.	3	(0.7)	3	(0.8)
Other forms of restlessness while asleep	Not at all.	411	(91.5)	326	(88.6)
	Yes, sometimes.	28	(6.2)	36	(9.8)
	Yes, frequently.	10	(2.2)	6	(1.6)

Regarding the results of the two-question case-finding instruments for depression, 141 (17.3%) athletes in total, 58 (12.9%) males and 83 (22.6%) females, replied “yes” to both questions (*p* < 0.01).

**Fig. 1:**
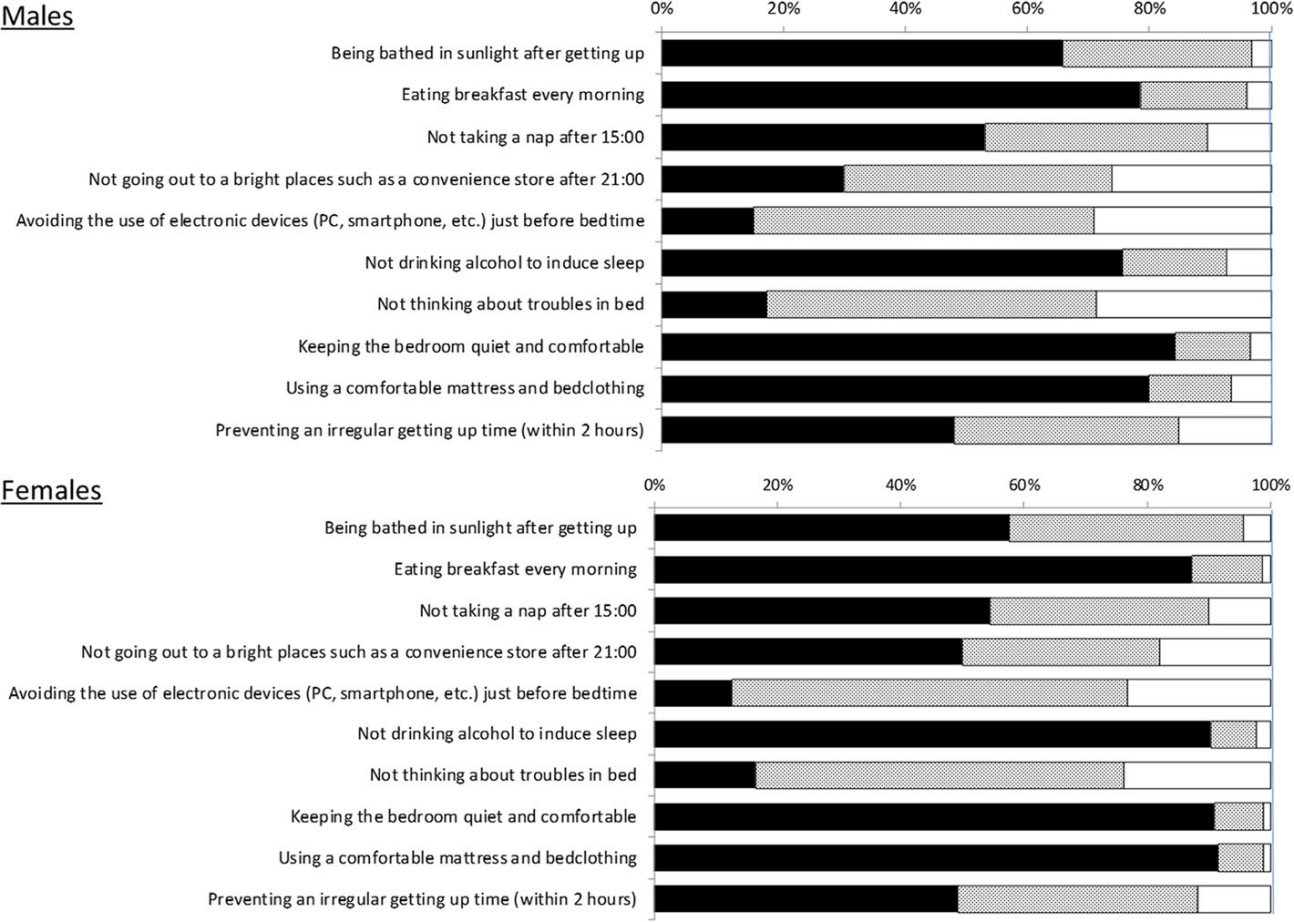
The results for sleep hygiene. ■: “I already do so.”, ⊡: “I may be able to, but do not already do so.”, and □: “I think it is difficult to do so”

The results of sleep hygiene are shown in Fig. 1. For both males and females, less than 20% of the athletes replied “I already do so” to the items “avoiding the use of electronic devices (PC, smartphone) just before bedtime” and/or “not thinking about troubles in bed.”

Table 5 describes the results of the univariate and multiple logistic regression analyses. The univariate logistic regression analysis revealed that sex, bed time, time in bed, depressive mood, and eight of ten sleep hygiene-factors were significantly correlated with poor sleep quality (PSQI global score > 5.5). In a multiple logistic regression analysis using the above variables, time in bed, depressive mood, and three sleep hygiene-factors were significantly correlated with poor sleep quality.

**Table 5:** The associations amongst sex, age, body mass index, experience of overseas trips, training-practice schedule, depressive mood and sleep habits, and poor sleep quality (PSQI global score > 5.5)

		β	SE	Wald-square	Odds	95% CI	*P* value
(A) Univariate logistic regression analysis							
General information	Sex	− 0.336	0.157	4.578	0.715	0.525–0.972	< 0.05
	Age	0.003	0.017	0.029	1.003	0969–1.038	0.86
	Body mass index	0.138	0.190	0.528	1.148	0.791–1.666	0.47
	Frequency of overseas trips	− 0.040	0.035	1.314	0.961	0.897–1.029	0.25
Daily schedule	Earliest training-practice start time	0.028	0.030	0.894	1.029	0.970–1.091	0.34
	Latest training-practice end time	− 0.055	0.039	1.952	0.947	0.877–1.022	0.16
	Hours of training-practice per week	− 0.006	0.007	0.780	0.994	0.980–1.008	0.38
	Bed time	− 0.222	0.083	7.200	0.801	0.682–0.942	< 0.01
	Getting up time	0.123	0.078	2.474	1.131	0.970–1.319	0.12
	Time in bed	− 0.357	0.081	19.586	0.700	0.598–0.820	< 0.001
	Nap duration	0.161	0.159	1.025	1.175	0.860–1.604	0.31
Clinical information	Loud snoring	0.105	0.122	0.743	1.111	0.874–1.412	0.39
	Sleep apnea	0.044	0.246	0.032	1.045	0.645–1.693	0.86
	Legs twitching or jerking during sleep	− 0.183	0.103	3.151	0.833	0.680–1.019	0.08
	Disorientation or confusion during sleep	− 0.215	0.186	1.341	0.806	0.560–1.161	0.25
Mood	Depressive mood	− 1.363	0.194	49.569	0.256	0.175–0.374	< 0.001
Sleep hygiene	Being bathed in sunlight after getting up	− 0.318	0.161	3.876	0.728	0.530–0.999	< 0.05
	Eating breakfast every morning	− 0.763	0.194	15.395	0.466	0.319–0.683	< 0.001
	Not taking a nap after 15:00	− 0.224	0.159	1.984	0.799	0.585–1.092	0.16
	Not going out to bright places after 21:00	− 0.150	0.164	0.831	0.861	0.624–1.188	0.36
	Avoid using electronic devices just before bed time	− 0.833	0.285	8.580	0.435	0.249–0.759	< 0.01
	Not drinking alcohol to induce sleep	− 0.462	0.197	5.506	0.630	0.429–0.927	< 0.05
	Not thinking about troubles in bed	1.066	0.166	41.021	2.903	2.095–4.023	< 0.001
	Keeping the bedroom quiet and comfortable	− 0.645	0.222	8.423	0.525	0.339–0.811	< 0.01
	Using a comfortable mattress and bedclothing	0.569	0.166	11.726	1.766	1.275–2.445	< 0.001
	Preventing an irregular getting up time (within 2 h)	− 0.614	0.163	14.276	0.541	0.392–0.744	< 0.001
(B) Multiple logistic regression analysis							
General information	Sex	− 0.147	0.188	0.608	0.863	0.597–1.368	0.44
Daily schedule	Bed time	0.099	0.109	0.823	1.104	0.891–1.368	0.36
	Time in bed	0.401	0.106	14.319	1.494	1.213–1.839	< 0.001
Mood	Depressive mood	− 1.170	0.212	30.496	0.310	0.205–0.470	< 0.001
Sleep hygiene	Being bathed in sunlight after getting up	− 0.166	0.185	0.802	0.847	0.589–1.218	0.37
	Eating breakfast every morning	− 0.593	0.225	6.955	0.552	0.355–0.859	< 0.01
	Avoid using electronic devices just before bed time	− 0.625	0.313	3.989	0.535	0.290–0.988	< 0.05
	Not drinking alcohol to induce sleep	− 0.174	0.224	0.606	0.840	0.541–1.303	0.44
	Not thinking about troubles in bed	− 0.759	0.184	16.962	0.468	0.326–0.672	< 0.001
	Keeping the bedroom quiet and comfortable	− 0.312	0.254	1.510	0.732	0.445–1.204	0.22
	Using a comfortable mattress and bedclothing	− 0.214	0.192	1.248	0.807	0.554–1.176	0.26
	Preventing an irregular getting up time (within 2 h)	− 0.328	0.184	3.182	0.720	0.503–1.033	0.07

The results of the univariate and multiple logistic regression analyses for males and females are described in Tables 6 and 7. For males, time in bed, depressive mood, and the hygiene-factor “not thinking about troubles” were significantly correlated with poor sleep quality (Table 6). For females, getting up time, depressive mood, the hygiene-factors “not thinking about troubles in bed” and “preventing an irregular getting up time (within 2 h)” were significantly correlated with poor sleep quality (Table 7).

**Table 6:** The associations amongst sex, age, body mass index, experience of overseas trips, training-practice schedule, depressive mood and sleep habits, and poor sleep quality (PSQI global score > 5.5) in males

		β	SE	Wald-square	Odds	95% CI	*P* value
(A) Univariate logistic regression analysis							
General information	Age	− 0.028	0.027	1.090	0.972	0.922–1.025	0.296
	Body mass index	0.006	0.037	0.024	1.006	0.935–1.081	0.878
	Frequency of overseas trips	0.029	0.047	0.381	1.029	0.939–1.129	0.537
Daily schedule	Earliest training-practice start time	0.000	0.040	0.000	0.995	0.925–1.082	0.995
	Latest training-practice end time	0.062	0.055	1.265	1.064	0.955–1.186	0.261
	Hours of training-practice per week	0.003	0.011	0.055	1.003	0.982–1.024	0.814
	Bed time	0.260	0.112	5.394	1.297	1.041–1.614	0.020
	Getting up time	0.027	0.103	0.067	1.027	0.840–1.255	0.796
	Time in bed	− 0.294	0.110	7.132	0.745	0.601–0.925	0.008
	Nap duration	− 0.039	0.220	0.031	0.962	0.625–1.482	0.861
Clinical information	Loud snoring	− 0.032	0.219	0.021	0.969	0.631–1.489	0.886
	Sleep apnea	− 0.291	0.470	0.383	0.748	0.298–1.879	0.536
	Legs twitching or jerking during sleep	0.292	0.219	1.772	1.339	0.871–2.059	0.183
	Disorientation or confusion during sleep	0.271	0.329	0.677	1.311	0.688–2.497	0.411
Mood	Depressive mood	1.667	0.294	32.198	5.297	2.976–9.421	0.000
Sleep hygiene	Being bathed in sunlight after getting up	0.430	0.224	3.695	1.537	0.992–2.384	0.055
	Eating breakfast every morning	0.851	0.241	12.761	2.366	1.475–3.796	0.000
	Not taking a nap after 15:00	0.173	0.219	0.621	1.188	0.774–1.825	0.431
	Not going out to bright places after 21:00	0.518	0.259	4.019	1.679	1.012–2.787	0.045
	Avoid using electronic devices just before bed time	0.873	0.396	4.853	2.394	1.101–5.206	0.028
	Not drinking alcohol to induce sleep	0.404	0.239	2.860	1.499	0.938–2.395	0.091
	Not thinking about troubles in bed	1.038	0.233	31.453	3.700	2.342–5.845	0.000
	Keeping the bedroom quiet and comfortable	0.781	0.264	8.778	2.184	1.303–3.663	0.003
	Using a comfortable mattress and bedclothing	0.721	0.224	10.391	2.056	1.326–3.187	0.001
	Preventing an irregular getting up time (within 2 h)	0.649	0.227	8.190	1.913	1.227–2.983	0.004
(B) Multiple logistic regression analysis							
Daily schedule	Bed time	0.108	0.137	0.623	1.114	0.852–1.456	0.430
	Time in bed	− 0.275	0.128	4.615	0.760	0.591–0.976	0.032
Mood	Depressive mood	1.497	0.319	21.976	4.468	2.390–8.355	0.000
Sleep hygiene	Eating breakfast every morning	0.431	0.284	2.308	1.539	0.882–2.685	0.129
	Not going out to bright places after 21:00	0.088	0.300	0.087	1.092	0.607–1.965	0.768
	Avoid using electronic devices just before bed time	0.552	0.443	1.555	1.737	0.729–4.138	0.212
	Not thinking about troubles in bed	0.952	0.257	13.776	2.591	1.567–4.284	0.000
	Keeping the bedroom quiet and comfortable	0.343	0.312	1.210	1.409	0.765–2.597	0.271
	Using a comfortable mattress and bedclothing	0.128	0.269	0.226	1.137	0.670–1.927	0.635
	Preventing an irregular getting up time (within 2 h)	0.188	0.269	0.486	1.207	0.712–2.046	0.486

**Table 7:** The associations amongst sex, age, body mass index, experience of overseas trips, training-practice schedule, depressive mood and sleep habits, and poor sleep quality (PSQI global score > 5.5) in females

		β	SE	Wald-square	Odds	95% CI	*P* value
(A) Univariate logistic regression analysis							
General information	Age	0.024	0.023	1.131	1.024	0.980–1.070	0.296
	Body mass index	0.047	0.043	1.191	1.048	0.964–1.139	0.878
	Frequency of overseas trips	0.007	0.050	0.022	1.007	0.914–1.111	0.882
Daily schedule	Earliest training-practice start time	− 0.063	0.045	1.918	0.939	0.859–1.026	0.166
	Latest training-practice end time	0.012	0.056	0.050	1.013	0.908–1.129	0.823
	Hours of training-practice per week	0.006	0.009	0.458	1.006	0.988–1.025	0.499
	Bed time	0.141	0.125	1.280	1.151	0.902–1.470	0.258
	Getting up time	− 0.277	0.127	4.794	0.758	0.591–0.971	0.029
	Time in bed	− 0.378	0.120	9.895	0.685	0.542–0.867	0.002
	Nap duration	0.361	0.233	2.411	1.435	0.910–2.265	0.121
Clinical information	Loud snoring	− 0.024	0.251	0.009	0.976	0.597–1.596	0.923
	Sleep apnea	1.061	0.772	1.888	2.889	0.636–13.121	0.169
	Legs twitching or jerking during sleep	0.336	0.227	2.183	1.399	0.896–2.185	0.140
	Disorientation or confusion during sleep	0.316	0.350	0.814	1.371	0.691–2.723	0.367
Mood	Depressive mood	1.036	0.257	16.218	2.817	1.702–4.663	0.000
Sleep hygiene	Being bathed in sunlight after getting up	0.187	0.225	0.693	1.206	0.776–1.873	0.405
	Eating breakfast every morning	0.848	0.301	7.908	2.334	1.293–4.214	0.005
	Not taking a nap after 15:00	0.343	0.224	2.342	1.409	0.908–2.186	0.767
	Not going out to bright places after 21:00	0.086	0.224	0.147	1.090	0.703–1.689	0.701
	Avoid using electronic devices just before bed time	0.772	0.410	3.546	2.164	0.969–4.835	0.060
	Not drinking alcohol to induce sleep	1.024	0.329	9.677	2.784	1.461–5.308	0.002
	Not thinking about troubles in bed	0.880	0.235	13.963	2.410	1.519–3.823	0.000
	Keeping the bedroom quiet and comfortable	0.624	0.340	3.369	1.865	0.959–3.630	0.066
	Using a comfortable mattress and bedclothing	0.454	0.234	3.776	1.574	0.996–2.489	0.052
	Preventing an irregular getting up time (within 2 h)	0.701	0.230	9.317	2.015	1.285–3.159	0.002
(B) Multiple logistic regression analysis							
Daily schedule	Getting up time	− 0.353	0.169	4.390	0.702	0.505–0.977	0.036
	Time in bed	− 0.173	0.154	1.257	0.842	0.622–1.138	0.262
Mood	Depressive mood	0.922	0.277	11.092	2.515	1.462–4.329	0.001
Sleep hygiene	Eating breakfast every morning	0.622	0.336	3.429	1.862	0.964–3.595	0.064
	Not drinking alcohol to induce sleep	0.652	0.356	3.359	1.920	0.956–3.857	0.067
	Not thinking about troubles in bed	0.692	0.254	7.403	1.997	1.213–3.286	0.007
	Preventing an irregular getting up time (within 2 h)	0.653	0.258	6.394	1.921	1.158–3.185	0.011

## Discussion

### The Prevalence and Factors Associated with Poor Sleep Quality

In the present study, we described the characteristics of the sleeping habits of elite Japanese athletes, investigated the prevalence of poor sleep quality on the PSQI global score, and explored its associated factors. Based on our knowledge, this is the first study to examine a large number of elite athletes (*n* = 817).

Our results using the PSQI global score indicated that sleep quality is poor in 28% of elite Japanese athletes. A previous study [36] that examined general populations in Japan reported that the PSQI global scores were 4.51 ± 2.14 for males in their 20s and 5.30 ± 2.48 for females in their 20s. This study also reported that 30.1% of males in their 20s and 36.4% for females in their 20s scored above the cutoff for poor sleep quality on the PSQI. Our results of the mean PSQI global scores and prevalence of poor sleep quality for both males and females were slightly better than those of age-matched general populations in Japan. The differences between sexes observed in the sleep schedules, PSQI global, and ESS scores in this study were in line with those in previous studies [36, 37].

The multiple logistic analysis revealed that athletes’ sleep quality was significantly associated with five factors: “time in bed,” “depressive mood,” “eating breakfast every morning,” “avoiding the use of electronic devices (PC, smartphone) just before bedtime,” and “not thinking about troubles in bed.” Regarding excessive daytime sleepiness, Doi and Minowa examined 4722 Japanese and reported that insufficient nocturnal sleep, an irregular sleep-wake schedule, and depression were associated risk factors [37]. Although the evaluated parameters differed between the latter research and ours (PSQI global and ESS scores), common background-related factors may exist.

### Time in Bed

Our study shows that the mean time in bed was 7 h and 29 min in total, corresponding to 7 h and 37 min for males and 7 h and 20 min for females. As shown in Table 2, males from five sports and females from nine sports showed a mean time in bed of less than 7 h. The 2015 NHK Japanese Time Use Survey revealed that the sleep duration was 7 h and 27 min for males in their 20s (*n* = 424) and 7 h and 18 min for females in their 20s (*n* = 437) [38]. Since sleep duration is generally shorter than time in bed, our results may suggest that time in bed of Japanese athletes was not as long as those of age-matched general populations. At present, it is unclear and debatable whether a time in bed that is the same as the general population is sufficient for athletes. However, some researchers recommend a longer nocturnal sleep, i.e., 8–10 h [39–41] for athletes. Samuels and Alexander [40] recommend 8–10 h of nocturnal sleep plus a 30-min nap between 2 and 4 pm for athletes. Bompa and Haff [41] reported that athletes require 9 to 10 h of sleep, 80–90% of it during the night. Rountree [42] recommends sleep extension with a longer training-practice time. Our results suggest that the time in bed of elite Japanese athletes is inconsistent with their recommendations.

Our study revealed that 34 (4.2%) athletes were considered short sleepers (time in bed < 6 h). Seventeen of the 34 short sleepers get up earlier than 6:00. However, only 4 of the 17 short sleepers reported that their training-practice starts before 8:00. Sargent et al. [4] reported that early-morning training markedly restricts the time in bed of swimmers. Our results differ from theirs. For an example, all of our kabaddi players reported that their training-practice starts around 19:00 and ends at 21:00. Twelve of 20 kabaddi players get up at 7:00 or earlier because of their jobs. For them, the schedules of both training-practice and their job may be the reasons for their short time in bed.

### Use of Electronic Devices

In this study, more than 80% of the athletes replied that they use electronic devices (PC, smartphone) just before bedtime. The Ministry of Posts and Telecommunications (Japan) reported that 53.7% of Internet users in Japan had delayed bedtimes, and 45.4% of them had a shortened sleep duration as a result of internet use [43]. It is also known that lights from electronic devices reduce melatonin levels, increase alertness at night, delay the circadian clock, and decrease alertness the next morning [22]. Thus, using electronic devices just before bedtime may lead to a shortened sleep duration and poor sleep quality [23–25].

### Skipping Breakfast

It is already known that skipping breakfast is associated with poor sleep quality [44]. Previous researchers have stated that busy lifestyles cause some people to skip breakfast, but some also suggested that skipping breakfast affects circadian rhythms. For example, in the liver, breakfast promotes food-induced entrainment of the circadian clock [45]. In addition, some researchers suggest that dietary components influence the synthesis of serotonin and melatonin, and this contributes to a good sleep quality [46]. Skipping breakfast may lead to the loss of these effects.

### Depressive Mood

Hammond et al. reported that 26% of athletes showed self-reported mild to moderate symptoms of depression [47]. In their study, they demonstrated that in elite athletes, negative changes in their performances were correlated with depression. Guilliver et al. examined 224 elite Australian athletes and reported that 27.2% of them displayed depressive symptoms [48]. Yang et al. examined 257 collegiate athletes and reported that 21% of their athletes showed depression, and anxiety and pain were related to that depression [49]. Other factors such as sports injuries, career termination, and performance outcomes that are below expectations are also risk factors of depression [50]. It is also well-known that a depressive mood is one of the symptoms of overtraining or overreaching [51]. Although it was unclear whether our athletes were suffering from overtraining or overreaching, such risks may arise during periods of high training loads [52].

Although sleep disturbance is known as one of the symptoms of depression, sleep deprivation lowers the psychological threshold for the perception of stress [53]. In addition, there is a report that individuals with short (< 7 h/night) and long (≧ 9 h/night) sleep durations show an increased genetic influence for depression [54]. Thus, sleep quantity and quality are associated with depression and vice versa.

### Thinking About Troubles in Bed

Symptoms of chronic stress, burnout, anxiety, and nervousness are prevalent amongst athletes [55]. Erlacher et al. studied 632 German athletes and reported that 65.8% of them experienced poor sleep during nights before important competitions [13]. Romyn et al. demonstrated strong negative associations between state anxiety and sleep quality in athletes [56]. Kashani et al. reported a significant correlation between perceived stress and the PSQI global score [57]. In non-pharmacologic treatment of insomnia, Lande and Gragnani recommend trying to resolve problems prior to bedtime or to make resolution a priority the following day [58]. Siebern et al. recommends relaxation techniques including progressive muscle relaxation, deep breathing techniques, body scanning, and autogenic training [59].

### Other Factors

Although the logistic regression analysis did not reveal a significant association with sleep quality, 40% of the athletes had been informed by someone about “snoring loudly” and/or “leg twitching or jerking during sleep” (Table 5). Similarly, Swinbourne et al. examined 175 athletes in New Zealand and demonstrated that 38% of them were defined as snorers and 8% reported having apnoeic episodes [18]. Tuomilehto et al. examined 107 professional ice-hockey players and found that 14 of them had obstructive sleep apnoea [19]. In our studies, loud snoring and a long pause between breaths were widely distributed in many BMI categories, and higher percentages of athletes were found in larger BMI categories. Emsellem and Murtagh said that a BMI above 28.0 kg/m^2^ and a neck circumference greater than 40.0 cm are risk factors for obstructive sleep apnoea syndrome [16]. However, this BMI criterion might be too high for the Japanese. Itasaka et al. divided 257 Japanese subjects into three groups: normal weight (BMI under 24.0 kg/m^2^), mildly obese (BMI 24.0–26.4 kg/m^2^), and obese (BMI 26.4 kg/m^2^ or higher), and showed that the apnoea-hypopnea index, intraoesophageal pressure, and lowest oxygen saturation became significantly worse according to the degree of obesity [60]. In this study, the athletes with a BMI at or above 25 kg/m^2^ showed a higher frequency of being informed about long pauses between breaths. However, some athletes with a BMI below 25 kg/m^2^ were also similarly informed. Other factors, such as microgenia, retrognathia, rhinostenosis, and enlarged tonsils, should also be examined to identify causes of apnoea/hypopnea. Regarding leg twitching or jerking, there are both physiological and pathological causes. If the leg twitching or jerking is physiological and does not fragment sleep, treatment is rarely necessary [61]. For pathological forms, sleep-related movement disorders, such as periodic limb movement disorder and restless legs syndrome, may be related. A low ferritin level (> 50 μg/L) is one of the risk factors of restless leg syndrome [62]. Koehler et al. reported that 31% of male athletes and 57% of female athletes showed serum ferritin levels below 35 μg/L [63]. Checking blood profiles might be recommended if sleep is disturbed by frequent limb movements. In a study on 107 professional ice hockey players, Tuomilehto et al. found 14 with obstructive sleep apnoea, 13 with insomnia, 4 with restless legs syndrome and periodic leg movements, 1 with parasomnia, and 1 with delayed sleep-wake syndrome [19]. We should keep in mind that factors not showing an association in our logistic analysis may also affect individual athlete’s sleep quality.

### Sex Differences

For both males and females, depressive mood and the hygiene-factor “not thinking about troubles in bed” were correlated with poor sleep quality (Tables 6 and 7). Time in bed for males and getting up time and the hygiene-factor “preventing an irregular getting up time (within 2 h)” for females were also correlated with poor sleep quality. These results suggest that psychological factors and sleep schedules are common factors that affect poor sleep quality.

### Limitations

Firstly, we investigated Japanese athletes’ sleeping habits using a questionnaire and not by actual measurements. If applicable, it would be desirable to evaluate sleep schedules with equipment such as actigraphy [2–4, 8, 9, 11, 12]. Secondly, Samuels et al. developed a new athlete-specific sleep-screening questionnaire because they felt that the PSQI classified a higher than expected number of athletes as poor sleepers [20]. For example, 24.7% of males and 32.1% of females showed a PSQI global score above 5.5, whereas our PSQI subcategory scores indicated that the number of athletes with insomnia seems to be small. It is debatable whether the PSQI is the most suitable sleep-screening questionnaire for athletes. Thirdly, athletes younger than 20 years old were excluded from this study. Since many of them spend the daytime at high school or junior high school, their training schedules and sleeping habits may be different from those shown by our results. Fourthly, we could not evaluate athletes’ performance in this research. Further studies are necessary to verify that an improvement in sleep leads to enhanced sport performance.

## Conclusion

In conclusion, 229 (28.0%) athletes had a PSQI global score showing poor sleep quality. Compared to the results in a previous study [37], our results suggest that the prevalence of poor sleep quality in athletes is slightly lower than that in age-matched general populations. The multiple logistic analysis in our study revealed that poor sleep quality on the PSQI global score is related to five factors: “time in bed,” “depressive mood,” “eating breakfast every morning,” “avoiding the use of electronic devices (PC, smartphone) just before bedtime,” and “not thinking about troubles in bed.” Forty percent of athletes reported they had been informed by someone about “snoring loudly” and/or “leg twitching or jerking during sleep,” which would suggest that sleep quality would be poor; however, this finding was not significantly associated with poor sleep quality on the PSQI global score. In the medical check for athletes, it may be desirable to include screening for sleep disorders.

## Abbreviations

BMI: Body mass index
ESS: Epworth Sleepiness Scale
PSQI: Pittsburgh Sleep Quality Index
